# Chronic Uterine Inversion in Post‐Menopausal Woman: A Rare Case Report

**DOI:** 10.1002/ccr3.72119

**Published:** 2026-02-25

**Authors:** Eyob Asefa Belay, Lami Gonfa Dinagde, Diriba Kebede Merga, Bezza Kedida Dabi, Tekle Wakjira Firisa

**Affiliations:** ^1^ Department of Obstetrics and Gynecology Nekemte Compressive Specialized Hospital Nekemte City Ethiopia; ^2^ Department of Obstetrics and Gynecology Wollega University Institute of Health Science Nekemte City Ethiopia; ^3^ Department of Obstetrics and Gynecology Jimma University School of Medicine Jimma Town Ethiopia

**Keywords:** Huntington's technique, hysterectomy, post‐ menopause, uterine inversion

## Abstract

Chronic uterine inversion is a rare disease presentation in obstetrics and gynecology discipline. It is the descent of uterine fundus inside out through the cervix. It is grossly classified as puerperal and non‐puerperal uterine inversion. Non‐puerperal uterine inversion is reported in 16% of all uterine inversions. Actual prevalence of puerperal uterine inversion is difficult to estimate in developing countries but estimated to be 1 in 20,000–50,000 cases. Chronic uterine inversion is commonly associated with other uterine pathology, mainly submucous uterine leiomyoma. Endometrial polyp, uterine sarcoma, previous repositioned inverted uterus and increased abdominal pressure are other associated factors. Abnormal vaginal bleeding, mass per vagina, foul‐smelling vaginal discharge and lower abdominal pain are frequent clinical symptoms. Uterine inversion is frequently misdiagnosed as uterine prolapse due to anatomical and symptom similarity. Physical examination, radiologic imaging and high index of suspicion help to diagnose and differentiate from uterine prolapse. Abdominal, vaginal or laparoscopic approaches are possible methods of treatment depending on the surgeon's experience. In abdominal and laparoscopic approaches, uterine repositioning prior to surgery is mandatory, either manual or surgical repositioning. Fifty years old, para 6, woman presented with chief complaint of mass per vagina of 3 months duration. It was associated with lower abdominal pain, vaginal bleeding and offensive vaginal discharge. She had intermittent low grade fever since the last one 1 month. On physical examination, she was tachycardic (pulse rate = 110) and febrile (temperature = 37.6°C centigrade). On HEENT examination, her conjunctiva was pale. On genitourinary system examination, there was offensive vaginal discharge, large mass protruding through vagina with 15 × 12 cm sized mass attached to uterus by broad pedicle. Overlying tissue was necrotic and fragile that bleeds easily. Cervix was not palpable. She was investigated with blood tests and abdominal ultrasound. Chronic uterine inversion with pedunculated leiomyoma severe anemia were diagnosed. After stabilizing the patient with transfusion and antibiotics, abdominal hysterectomy was done after uterus placed into abdomen surgically, as manual repositioning was difficult. Haultain's technique was used to reposition uterus into abdomen. Post‐operative course was smooth and she was discharged home with improvement. Two weeks after discharge, she visited gynecologic out‐patient department and it was smooth course. Chronic uterine inversion is a rare disease. It is grossly classified as puerperal and non‐puerperal uterine inversion. Pelvic pathologies like leiomyoma, endometrial polyps, uterine neoplasms, and large bladder stones are predisposing factors. It is frequently misdiagnosed as uterine prolapse. Frequently, physical examination, common radiologic imaging like ultrasound, and a high index of suspicion are adequate to reach a diagnosis. In limited cases, like grade 1 and 2 uterine inversion, patients with no risk factors and adolescents, advanced diagnostic imaging like MRI and CT scans are recommended. Surgical intervention is the primary management, either for uterine preservation or hysterectomy. Although there is no recommended standards of operation approach, Adnominal, laparoscopic or vaginal approaches are possible option of surgical route depending on surgeon's experience. Chronic uterine inversion is a rare disease entity. It has diagnostic and treatment challenges. It is commonly misdiagnosed as uterine prolapse. A high index of suspicion is crucial during the evaluation of a patient presenting with protrusion of mass per vagina as uterine prolapse mimics uterine inversion. There is no standardized surgical management. Surgical approach, either abdominal. Vaginal or laparoscopic, depends on surgeon's experience.

## Key Clinical Message

Chronic uterine inversion is a rare disease entity but an important cause of vaginal mass in post‐menopausal women, often misdiagnosed as uterine prolapse. Frequently, it is associated with uterine leiomyoma, uterine sarcoma, endometrial polyp, and bladder stone. Clinicians should maintain a high index of suspicion and use imaging when needed. Surgical management is a mainstay treatment. Uterine repositioning, either vaginal or abdominal, is required for safe hysterectomy.

## 1 Introduction

Chronic uterine inversion is a rare disease presentation in obstetrics and gynecology discipline which is seen in wide range of reproductive age group, rarely in adolescent and frequently in post‐menopausal age [1]. Uterine inversion is descent of uterine fundus inside out through the cervix [2, 3]. It descends either partially, mere dimpling of uterine fundus, or fully, complete inversion of uterine through the cervix [4]. It is grossly classified as puerperal and non‐puerperal uterine inversion [3]. Puerperal uterine inversion is most common type uterine inversion, 85.8% cases, and associated with higher rate maternal mortality and morbidity [2]. Maternal death results from neurologic and hemorrhagic shock due to parasympathetic nerves system stimulation and uterine atony. Prevalence of puerperal uterine inversion is reported 1 in 20,000–50,000 cases [5]. It affects wide range of reproductive age group, adolescent to post‐menopausal [6]. Actual prevalence of puerperal uterine inversion is difficult to estimate in developing countries due to high rate of home delivery in hand of untrained birth attendant. It is caused due to mismanagement of third stage of labor management [3, 4]. Non‐ puerperal uterine inversion is reported in 16% of all uterine inversion [2, 7]. It is classified as subacute (48 h post‐partum—4 weeks) and chronic inversion (greater than 4 weeks of post‐partum) [8].

Chronic uterine inversion is frequently associated with uterine pathology, mainly submucous uterine leiomyoma [7]. Other predisposing pelvic pathologies are endometrial polyps, uterine neoplasms, and large bladder stones, previous operative procedures to reposition the uterus [7, 9, 10]. Increased intra‐abdominal pressure and post‐menopausal hormonal replacement are promoting factors [2]. It is classified as stage 1: the uterine fundus is inverted and the uterine fundus is located in the uterine cavity; stage 2: the uterine fundus is inverted and the fundus is located in the vaginal cavity; stage 3: the uterus is completely inverted with the uterine fundus protruding beyond the vaginal orifice; and stage 4: the uterus is completely inverted and is prolapsing out of the vulva through the vagina [3, 8]. Frequently, it is misdiagnosed as uterine prolapse due to the anatomic and clinical symptoms' similarity. Abnormal vaginal bleeding, protrusion of mass per vagina, foul smelling vaginal discharge, abdominal pain, and urinary incontinency are frequent clinical presentations of chronic uterine inversion [7].

Diagnosis of chronic uterine inversion mainly depends on clinical history, physical examination and imaging, mainly ultrasound. There is a diagnostic challenge in grade 1 and 2 which may need advanced imaging like MRI and CT scan [1, 11]. Surgical intervention is the primary management, either for uterine repositioning or hysterectomy. Uterine preservation or hysterectomy is decided based on the patient's need for fertility and associated complications such as uterine necrosis, presence of malignancy and superinfection [9]. Prior to proceeding, whether uterine preservation or hysterectomy, repositioning of the uterus into the abdomen is mandatory except in some cases. There are two techniques of uterine repositioning, manual and surgical. Manual uterine repositioning is first tried and if it is difficult or fails, surgical repositioning will be done. There two surgical repositioning approaches, abdominal approach (Haultain's or Huntington's technique) and vaginal approach (Kustner's and spinelli's technique) [5, 6, 8]. The type of surgery to be performed is determined by the patient's fertility need and any associated complications such as uterine necrosis and suspected malignancy. The surgical approach is either abdominal, laparoscopic, or vaginal [5, 12]. Currently, there is a standardized surgical route approach and it largely depends on the surgeon's experience. Hysterectomy is performed when a woman does not need fertility and any associated complications that preclude uterine preservation [1, 11]. As chronic uterine inversion is rare that even some gynecologists did not see a single case in their lifetime [11, 13]. We report this case because of its rarity, diagnostic and treatment challenge of chronic uterine inversion. We believe that the approach we followed to diagnose and treat the patient will add knowledge to the medical and public health community.

## 2 Case Presentation

### 2.1 History

A 50 years old, para six, woman presented to wollega university gynecologic outpatient department with chief compliant of protrusion of mass per vagina of 3 months duration which progressively increased in time. It was associated with lower abdominal pain which is moderate in severity and non‐radiating pain since protrusion of the mass. She developed vaginal bleeding and offensive vaginal discharge since the last 1 month. She has intermittent high grade fever. She has six alive children and gave birth all vaginally. She has palpitations and light headedness. She is referred from surrounding health center with a diagnosis of pelvic organ prolapse. She arrived to hospital supported by family. She has no chronic medical illness.

## 3 Pertinent Physical Examination

**FIGURE 1:**
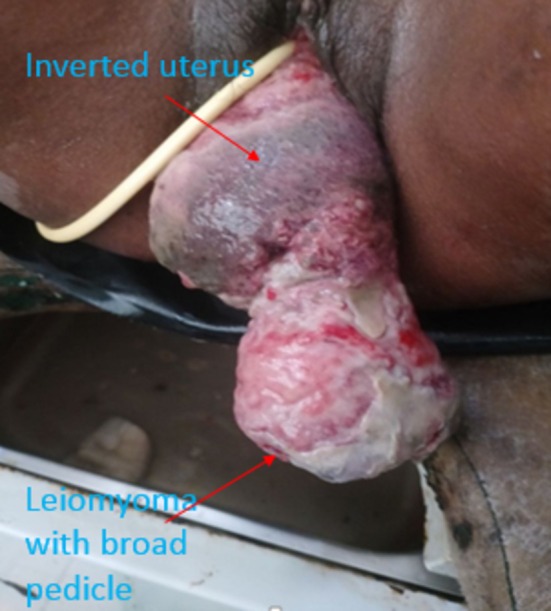
Large leiomyoma attached to fundus of inverted uterus prior to operation.

Finding on vital signs were pulse rate 110 beats per minute (tachycardic), blood pressure 100/60 mm of mercury (normal), temperature 37°C centigrade and respiratory rate 24 breaths per minute. On head and neck examination, her conjunctiva was pale. Her chest was clear and resonant. On cardiovascular examination, S1 and S2 were well heard and there was no gallop nor murmur. Pertinent examination was on genitourinary system examination. Cervix was not palpable. A large mass, measuring 15 × 12 cm that attached to the uterine fundus with a broad pedicle was seen. Figure 1, shows a large leiomyoma attached to the uterine with a broad pedicle. Overlying tissue was necrotic and easily bleeds with contact. There was offensive vaginal discharge. Abdomino‐pelvic ultrasound revealed that the uterus was not visualized in the abdomen. There was no abdominal collection. Kidneys, liver, and urinary bladder were normal and index diagnosis was uterine prolapse. On complete blood count, WBC was 15,000 with neutrophil predominance, 82%, and hemoglobin was 6.5 g/dL. Urinalysis was normal and HIV test was negative.

## 4 Differential Diagnosis

– Pedunculated leiomyoma
– Leiomyosarcoma
– Endometrial polyp
– Bladder cancer
– Incarcerated uterine prolapse

With above mentioned history, physical examination and laboratory tests, diagnosis of infected chronic uterine inversion secondary to leiomyoma and severe anemia were diagnosed. Patient was admitted to ward and started on broad spectrum antibiotics. She was transfused two unit of whole blood to correct anemia. Necrotic tissue was debrided. Pathology specimen from the mass was sent and confirms that it was leiomyoma with necrotic tissue. After correction of anemia, she was scheduled for operation. The first step of operation was repositioning of uterus into the abdomen. After the uterus and mass were cleaned with povidone iodine, assistant tried to reposition the uterus into the abdomen manually. Then we decided to reposition the uterus surgically Haultain's technique and it was successful. After ureters were identified bilaterally, extra fascial hysterectomy was done. Intra‐operative and post‐operative courses were smooth. The patient was discharged home after 7 post‐operative days of hospital stay. The visited our follow up clinic after 14 days of discharge and there was no problem.

## 5 Discussion

Uterine inversion is the descent of the uterine fundus inside out through the cervix, either partially or fully. It is grossly classified as puerperal and non‐puerperal uterine inversion [11]. Puerperal uterine inversion is the most common type and is associated with a higher rate of maternal mortality and morbidity. Chronic uterine inversion is commonly associated with other uterine pathology, mainly submucous uterine leiomyoma [13, 14]. Other pathologies like endometrial polyps, uterine neoplasms, and large bladder stones are possible predisposing factors [1]. In our case, chronic uterine inversion was associated with large pedunculated sub serous leiomyoma with overlying necrotic tissue which is similar to case reports from saint Paul's millennium hospital, Ethiopia and King Abdul Aziz Hospital, Riyadh, Saudi Arabia [15, 16]. Chronic uterine inversion is one of the rare disease presentations which is seen in a wide range of reproductive age groups, rarely in adolescents and frequently in post‐menopausal age. In our case, she is a post‐menopausal woman, presented with vaginal bleeding and a mass of 3 months duration. Uterus was incarcerated and superinfected. Pathology has confirmed that there was no malignancy. Necrotic tissue was debrided and she was started on broad spectrum antibiotics.

Frequently, patients with chronic uterine inversion present with abnormal vaginal, offensive vaginal discharge, mass protrusion per vagina, lower abdominal pain, and urinary symptoms. Overlying tissue of protruded mass is commonly necrotic. On physical examination, cervix is not visible or palpable. In our case, the patient presented with abnormal vaginal bleeding and protrusion of mass of 3 months duration. She has urinary frequency and offensive vaginal discharge too. These clinical presentations are similarly reported in case reports from Gauhati Medical College, Guwahati, India and Ahmadu Bello University Teaching Hospital, Nigeria [7, 13].

Diagnostic challenge arises in grade 1 and 2 uterine inversion. Grade 3 and 4 uterine inversions are visible through vagina and physical examination finding could suggest uterine inversion but frequently misdiagnosed as uterine prolapse [13, 17]. Frequently, physical examination and ultrasound are diagnostic. MRI and CT scan are requested in case there are other associated factors like pelvic or abdominal tumors, grade 1 or 2, early reproductive age and cannot reach a diagnosis [13, 17]. A case report from National Guard hospital, Riyad, had indicated that CT scan is important diagnostic imaging in grade 2 uterine inversion and associated pelvic mass. It had showed that CT scan clearly shows course of ureter in relation to inverted uterus and pelvic mass [16]. In our case, diagnosis was reached by physical examination and ultrasound. On physical examination, cervix was not palpable and uterus was not visualized on pelvic ultrasound study. At presentation, it was grade 4 uterine inversion, which is shown on Figure 1 and large pedunculated sub‐mucous leiomyoma was visible attached to uterine fundus. In case reports from Ethiopia and India have revealed that chronic uterine inversion diagnosis can be reached with physical examination and ultrasound study [7, 11].

**FIGURE 2:**
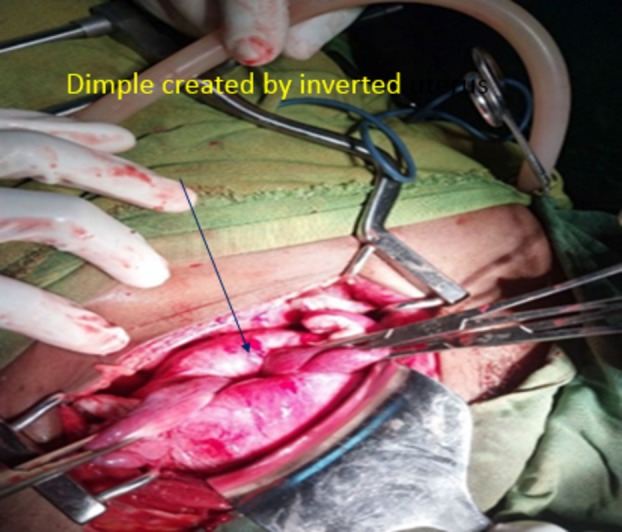
Intraoperative finding, dimple created by inverted uterus prior to repositioning.

**FIGURE 3:**
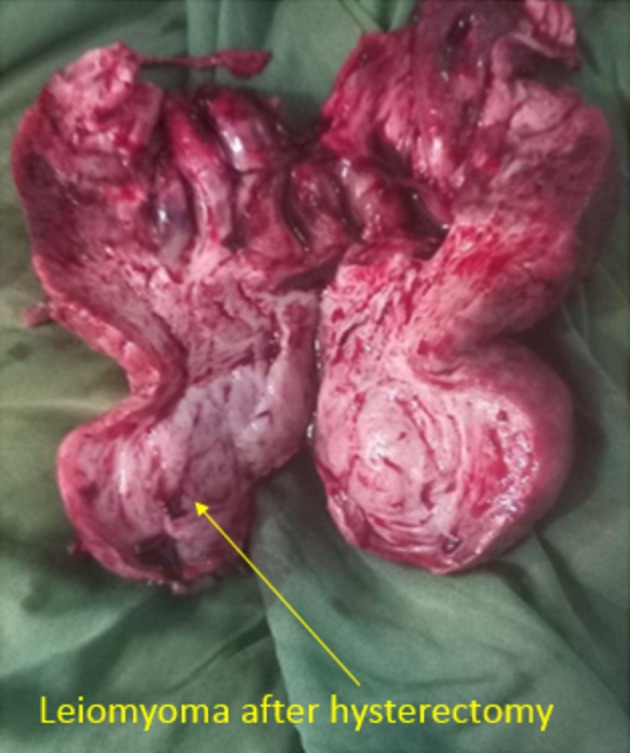
Gross appearance of leiomyoma and uterus after hysterectomy was done and specimen dissected.

Surgical intervention is primary management, either repositioning of the uterus or hysterectomy based on need for fertility and associated complications such as uterine necrosis [17]. Surgical approach of chronic uterine inversion is either laparoscopic or open surgery. Prior to proceeding, whether uterine preservation or hysterectomy, repositioning of the uterus into the abdomen is safe and practical. Manual uterine repositioning is first tried and if it is difficult or fails, surgical repositioning will be done [8]. There are two approaches of surgical repositioning, abdominal approach (Haultain's or Huntington's technique) and vaginal approach (Kustner's and Spinelli's technique) [12, 14, 17]. In some patients, uterine repositioning into the abdomen can be difficult due to large leiomyoma attached to the uterus. In such cases, combined vaginal surgery, to reduce mass, and abdominal surgery to do hysterectomy, can be preferred. One case report from Ethiopia had stated that combined vaginal and abdominal surgery were done due to the difficulty of large mass replacing into the abdomen [15]. Patient was counseled on surgical management options and she opted for hysterectomy. In our case, she is a post‐menopausal woman and due to high risk of recurrence, we decided on hysterectomy too. Up on laparotomy, we confirmed uterine inversion as shown on Figure 2. We tried to reposition the uterus into the abdomen manually but failed. Uterus reposition was successful with Haultain's technique. Then, we proceeded with abdominal hysterectomy. Ureter injury rate is expected to be high and we identified and stented ureters bilaterally. Specimen was dissected and revealed gross appearance of leiomyoma which is indicated in Figure 3. Intraoperative and post‐operative conditions were smooth. A case report from Jima University, Ethiopia, showed that in some patients it is difficult to reposition the uterus into the abdomen even with abdominal surgical techniques. They failed to reposition the uterus both by Huntington's and Haultain's technique, and proceeded with vaginal hysterectomy with direct visualization of the ureter through laparotomy opening. This indicates that uterine repositioning into an anatomic state is mandatory for safe surgery even though vaginal hysterectomy is possible [2]. Our patient was appointed after 2 weeks of surgery for follow‐up. She visited the gynecologic outpatient department in the second week and her post‐operative condition was smooth.

## 6 Conclusion

Chronic uterine inversion is a rare disease entity. In post‐menopausal women presenting with vaginal mass and bleeding, chronic uterine inversion should be considered. Frequently, it is associated with uterine pathology like uterine leiomyoma, endometrial polyp, and uterine sarcoma. It has diagnostic and treatment challenges specifically in grade 1 and 2, associated factors like malignancy and pelvic mass. Uterine preservation or hysterectomy are options of management. There are no standardized surgical management options but safe management requires uterine repositioning into the abdomen prior to abdominal hysterectomy. Vaginal, abdominal, or laparoscopic approaches solely depend on the surgeon's experience.

## Author Contributions

**Eyob Asefa Belay:** conceptualization, data curation, investigation, methodology, writing – original draft, writing – review and editing. **Lami Gonfa Dinagde:** conceptualization, data curation, investigation, methodology, writing – original draft, writing – review and editing. **Diriba Kebede Merga:** investigation, methodology, writing – original draft, writing – review and editing. **Bezza Kedida Dabi:** investigation, methodology, writing – original draft, writing – review and editing. **Tekle Wakjira Firisa:** writing – original draft, writing – review and editing.

## Funding

The authors have nothing to report.

## Consent

Informed written consent was obtained from the patient for the images and potential publication of the case in accordance with the journal's patient consent guideline.

## Conflicts of Interest

The authors declare no conflicts of interest.

## Data Availability

Data used during this study is available with the corresponding author upon reasonable request.
